# Context-dependent dynamic UV signaling in female three spine sticklebacks

**DOI:** 10.1038/srep17474

**Published:** 2015-12-10

**Authors:** Meike Hiermes, Theo C. M. Bakker, Marion Mehlis, Ingolf P. Rick

**Affiliations:** 1 Institute for Evolutionary Biology and Ecology, University of Bonn, An der Immenburg 1, 53121 Bonn, Germany

## Abstract

Color signals, including ultraviolet (UV) signals, are widespread throughout the animal kingdom and color changes can be influenced by reproductive and motivational state. However, studies on dynamic changes of UV signals are scarce. Three spine sticklebacks (*Gasterosteus aculeatus*) that show intraspecific UV communication were used to study dynamic UV signaling in females. Reflectance measurements were taken from the distended abdomen, which serves as signal of female fecundity and readiness to spawn for courting males, and the melanized dorsal region. Scans were taken during egg maturation as well as before and after stimulation with a male to investigate context-dependent color changes. We used a physiological model of vision to determine how females might be perceived by conspecifics and quantified chromatic contrasts among both body regions and between body regions and the background for all stages. Females showed a significant increase in abdominal UV intensity during egg maturation and in response to a courting male. Measures of chromatic contrast among body regions (abdomen vs. dorsal region) and against the background (abdomen vs. background) were also increased during egg maturation and in response to the male stimulus (abdomen vs. background). Our results provide evidence for dynamic UV signaling in females in a reproductive context.

Throughout the animal kingdom manifold elaborate ornamental traits can be found, which are often the result of sexual selection^1^. Fishes show an extraordinarily huge variation in coloration^2^. The diversity of colors found in nature is based on complex, three-dimensional structures, which often contain multiple pigment types or have different structural properties. Two types of chromatophores (color cells) are distinguished: pigmented and structural cells. There are five pigmented chromatophore types in fish: melanophores (black color), xanthophores (yellow color), erythrophores (red color), cyanophores (blue color) and leucophores (white color)^2^,^3^. Iridophores (metallic or iridescent color) are the only structural color cell in fish and responsible for the silvery sheen of many fish species^4^. These structural cells, unlike pigment-based cells that work on the basis of pigment granules moving towards the cell periphery to produce the respective colors, contain guanine crystals that produce different colors depending on arrangement^2^,^3^.

Color signals are most often dynamic and may vary depending on context^5^ and color change can be seasonal or ephemeral^6^. Seasonal color changes are promoted morphologically by changing the number of chromatophores^7^, while ephemeral color changes (physiological color change) can be accomplished very rapidly either through an aggregation or dispersal of the pigment-containing organelles within the chromatophores^8^,^9^,^10^ or through a change of reflective capacities of iridophores^11^,^12^,^13^,^14^,^15^,^16^. Conspicuous coloration and color changes are widespread during the reproductive season, and often changes are influenced by factors such as body condition, motivational state, social status or reproductive status and intention during courtship and vary with respect to structure and time^6^.

The development of a conspicuous courtship coloration in general is most often attributed to males^1^, however, female coloration is widespread^17^,^18^,^19^,^20^,^21^,^22^. Courtship coloration has been shown to be dynamic, e.g. during certain stages of the breeding cycle, and may as well be enhanced or diminished^23^,^24^,^25^ to signal aspects of the female reproductive status or female quality^26^.

The three spine stickleback (*Gasterosteus aculeatus*), used as model organism in this study, is a small cold-water fish exhibiting a pronounced sexual dichromatism during the reproductive season. Outside the breeding season males and females are mottled black on a silvery-gray ground, although there exists genetic variation of patterns between populations^27^. At the beginning of the breeding season males develop a characteristic carotenoid-based red-orange throat coloration^28^,^29^. In contrast, breeding females are characterized by a silvery flank and abdominal coloration^30^. The silvery, egg-swollen abdomen, which is associated with female spawning-readiness and fecundity, is presented to the male during courtship in a so-called “head-up” posture^31^,^32^. In simultaneous choice tests stickleback males preferred females in the “head-up” posture over those in horizontal orientation^33^. Furthermore, when males were provided with females showing different degrees of abdominal extension, they showed a preference for the more distended ones^31^,^34^,^35^, demonstrating that the swollen belly is a signal of particular importance in male mate-choice in this species. In some populations reproductively active females also develop a dark-blackish bar-pattern on dorsum, flanks and tail stem^24^,^36^, which was preferred in male mate-choice experiments^24^. Beyond that, irrespective of the previously mentioned color signals in the human-visible part of the light spectrum, the sticklebacks’ visual system is based on four cone types, one well within the UV spectral range (λ = 360 nm)^37^. Accordingly, all stickleback populations tested thus far are reflective in the UV spectral region, which accounts for both sexes^36^,^37^,^38^. The silvery, distended abdomen of gravid stickleback females shows a reflectance peak in the UV spectral range providing a contrast to melanized dorsolateral regions with only low UV reflectance^36^. The short-wave UV signals have been shown to be of importance in female^39^,^40^ and male^36^ mate-choice. In detail, gravid females presented under UV-present conditions were preferred by males compared to individuals shown under UV-absent conditions^36^.

Several studies have dealt with the dynamic expression of male courtship signals in the human-visible wavelength range indicating temporal changes in the red mosaic signal^41^,^42^,^43^. Moreover, changes in sensitivity for red coloration with changing reproductive status have been reported for both stickleback sexes^44^,^45^,^46^. However, the dynamics of UV signals have been overlooked thus far and nothing is known about changes in UV signaling during the egg-ripening cycle of females as well as about short-term dynamics of UV signaling. Overall, in comparison to studies focusing on male color changes, only a limited amount of studies have taken dynamics of female coloration^23^,^24^,^25^ and the dynamics of signals in the UV spectral range into account^12^,^13^,^47^,^48^,^49^,^50^. A recent study by White *et al.*^51^ on the butterfly *Hypolimnas bolina* showed dynamics in male UV coloration of the dorsal wing that were not dependent on changes in structural coloration, but on changes in viewing angle during courtship. Kasukawa *et al.*^12^ and Mäthger *et al.*^13^ demonstrated immediate color changes in the UV spectral region, while Ornborg *et al.*^47^, for example, showed seasonal changes in UV structural color for both sexes in the blue tit (*Pavus caeruleus*). However, the studies dealing with the dynamics of UV signals did not explicitly consider short-term changes in a reproductive context.

In the present study, we thus tested for color changes in the UV spectral range both during the egg-ripening cycle of female three spine sticklebacks and for short-term changes in coloration in response to a standardized computer-animated male in an intersexual courtship context.

## Methods

### Ethics statement

The study conforms to the Association for the Study of Animal Behaviour Guidelines for the use of animals in research as well as to the legal requirements of Germany. The parental generation of the F1 sticklebacks used in this study was purchased from a commercial fisherman (Texel, the Netherlands), who has the permission to catch the fish. The study was approved by the Deutsche Forschungsgemeinschaft (DFG) (Project No. BA 2885/1-5). No further licenses were needed.

### Experimental subjects

Three spine sticklebacks of an anadromous population were caught during their spring migration in April 2011 on the island of Texel, the Netherlands. Fish from this population possess UV vision as they discriminated between light habitats differing only in UV spectral content^52^. Test fish were the F1 generation of random crosses of these wild-caught ancestors. Eggs were laid between May and August 2011 and were taken out of the males´ nests shortly after fertilization. In total, 26 families were produced. Each clutch was kept in an 1-l plastic box aerated with an airstone and illuminated by fluorescent tubes, which provided light with a proportion of UV similar to natural daylight (Truelight, T8/18W, T8/36W, T8/58W) in an air-conditioned room (17 ± 1 °C) under summer conditions (day/night 16 h/8 h). All fish were fed to excess with *Artemia* nauplii until they were 20 weeks old. At 20 weeks of age fish were transferred to larger holding tanks measuring L 50 cm × W 30 cm × H 30 cm, equipped with an internal filter and were fed from thereon to excess with frozen mosquito larvae (*Chironomus* spec.). The light regime was changed to winter conditions (day/night 8 h/16 h) on October 15^th^ 2011 and on June 15^th^ 2012 light conditions were changed back to a standardized summer light regime (day/night 16 h/8 h) in order to simulate the beginning of the breeding season four weeks prior to experiments.

### Experimental design

Two different experiments were conducted to test whether UV color signals of stickleback females vary during the course of an egg-ripening cycle (experiment 1) and as short-term response to an intersexual stimulus, before and after being visually exposed to a standardized computer-animated courting male (experiment 2).

#### (1) Egg-ripening cycle

**Figure 1:**
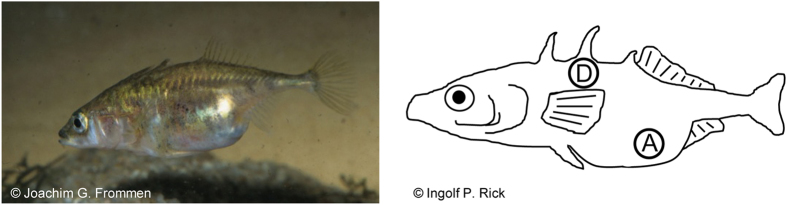
Receptive stickleback female. Reflectance measurements were taken in the abdominal region (**A**) and in the melanized dorsal region below the second spine (**D**).

Receptive females were caught from their holding tanks and were then stripped of all eggs. Right after stripping, reflectance measurements were conducted at the abdomen and in the melanized dorsal region below the second spine (see Fig. 1 and below for details). Afterwards females were moved to individual tanks (30 cm × 20 cm × 20 cm). Reflectance measurements were continued every second day until females were ripe again. Three measuring points were later taken into account: a) after stripping, b) during the ripening process (as the mean of all measurements between stripping and being ripe again), and c) being ripe. In total, sixteen females were tested and families were never used twice.

### (2) Short-term response

Stickleback females react to multiple visual traits of potential mating partners (e.g. body size^53^, symmetry^54^ and red throat^55^). We thus used a computer animation to standardize tests on the influence of an intersexual stimulus on female color expression. Computer animations work very well in sticklebacks and have been used frequently^54^,^56^,^57^. The computer-animated male stimulus was adopted from a previous study^56^ and the video colors of the stimulus presentation were modified according to the spectral characteristics of the ‘natural’ red breeding signal from reproductively active males as perceived through the stickleback visual system^58^,^59^,^60^. The RGB values (R = 238, G = 61, B = 8) assigned to the red courtship coloration of the artificial male correspond to intensely red colored males from the present study population. The basic computer animation used was constructed by Künzler & Bakker^56^ and lasts 150 seconds in total. The sequence starts with the display of a gray-colored landscape (5 s), followed by the entrance of the computer-animated red-colored male on the left, which shows fanning and zig-zagging movements (courtship behavior) (28 s). The whole sequence is repeated four times after which the male leaves the scene and an empty background is visible again^58^.

At first, ripe females (N = 23, families were never used twice) were gently netted from the holding tanks and were isolated in single aquaria (30 cm × 20 cm × 20 cm). The following day, reflectance measurements were conducted at the distended UV-reflecting abdomen and in the melanized dorsal region below the second spine (see Fig. 1 and below for details). Right afterwards females were transferred back to their home aquarium, which also served as experimental aquarium to reduce stress due to handling for the females. The aquarium was positioned in front of a computer monitor (ViewSonic G90fB, Model VS10794) and was elevated to match the height of the computer screen. The screen was covered up by black wall paper, so that the fish was only able to view the computer animation through a 10 cm × 6 cm viewing window cut into the black paper but not the rest of the screen. The animation was controlled via a laptop (Fujitsu Siemens V5535). The lighting conditions (illumination provided by Truelight T8/36W fluroescent tubes) during testing resembled those during rearing and during the short isolation phase of the females. Fish were allowed to acclimatize for ten minutes, during which the empty landscape was visible on the computer screen. After this acclimation time, the computer animation was started and repeatedly shown for ten minutes. Afterwards, reflectance measurements were taken again.

### Reflectance measurements

**Figure 2:**
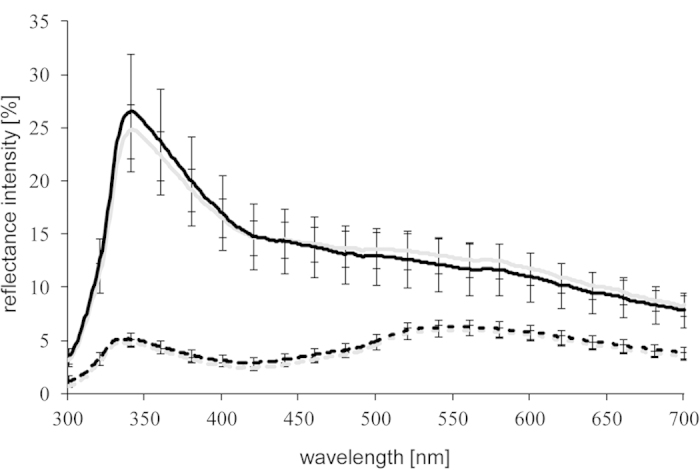
Reflectance spectra of body regions. Mean reflectance (proportion of light reflected in relation to a white standard (see text)) in the abdominal region (solid lines) and in the dorsal region (dashed lines) taken before (gray lines) and after (black lines) the presentation of a computer animation of a reproductively active male. Given are means ± standard errors.

**Figure 3:**
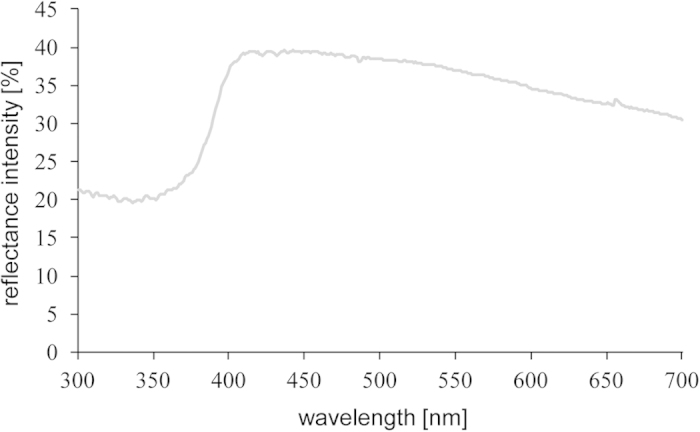
Reflectance spectrum of the visual background. Mean reflectance (proportion of light reflected in relation to a white standard (see text)) of the visual background used during rearing and in the experimental set-up.

All reflectance measurements were taken using an Avantes AvaSpec 2048 fiber-optic spectrophotometer in the abdominal region and in the dorsal region below the second spine (see Fig. 1). The dorsolateral bar-like pattern found in some stickleback populations^24^ was absent in our study population. Light was provided by a deuterium-halogen-light source (Avantes AvaLight-DHS Deuterium-Halogen Light Sources, 200–1100 nm). Reflectance scans were taken relative to a 98% Spectralon white standard (300–700 nm) and conducted with a bifurcated 200 micron fiber-optic probe and a fitted black cap (angle: 45°), which was held to the body surface in a distance of 3 mm. The software Avantes AvaSoft 7.5 was used to record individual scans (20 per measurement), which were exported to Microsoft Excel (see Fig. 2 for mean reflectance spectra of ripe females). Using the same measurement protocol, we measured the spectral reflectance from the visual background in the stimulus compartments, which consisted of gray plastic partitions (Fig. 3). We used visual modeling to evaluate how sticklebacks might be perceived by conspecifics. In a first step, we determined the spectral sensitivity curves for the four stickleback cone receptors from cone absorbance maxima provided in Rowe *et al.*^37^ and by using parameters for the calculation of visual pigment templates provided in Govardovskii *et al.*^61^. Subsequently, using Avicol_v6^62^, by multiplying individual reflectance, the ambient light (spectrum of the fluorescent tubes used during rearing and experiments (Truelight T8/36W)) and the calculated spectral cone sensitivities, we calculated absolute cone stimulations (UV, S, M, L)^63^,^64^. Absolute cone stimulations were then converted to relative cone stimulations and translated to the Cartesian coordinates x, y and z. These Cartesian coordinates were then converted to three spherical coordinates (*theta*, *phi*, chroma *r*), which define a color vector within a tetrahedral color space^63^,^64^,^65^. Within the tetrahedral color space, the central point is the achromatic point of black, white or gray color^65^,^66^. The color intensity (chroma *r*) is defined as the distance of the achromatic point from a given color point (defined by the angles *phi* and *theta*, representing hue). The larger the magnitude of chroma, the larger is the distance from the achromatic point and thus the higher is the color intensity. We used achieved chroma *r*_A_ as a measure of color intensity, which is the value for chroma *r* in comparison to the maximum possible value of for a specific hue (*r/r*_*max*_)^65^,^66^. Due to the one-peak nature of the reflectance spectra in the UV spectral region (Fig. 2; abdominal region), achieved chroma *r*_A_ is here used as measure for color intensity in the UV waveband. To allow for better comparison with other studies, we furthermore calculated the colorimetric variable “UV chroma”, which is independent from the receiver’s visual system and corresponds to achieved chroma *r*_A_ obtained by the physiological model. To determine “UV chroma”, a measure of the relative intensity in the UV spectral range between 300 nm and 400 nm was calculated relative to the total amount of light in the spectral range between 300 nm and 700 nm^38^,^67^. UV chroma and achieved chroma *r*_A_ were highly significantly correlated (Pearson’s product-moment correlation; egg-ripening cycle: t = 7.017, df = 46, p < 0.001; short-term response: t = 12.110, df = 44, p < 0.001).

Furthermore, we calculated the chromatic conspicuousness (i.e. chromatic contrast) as the Euclidean distance between the chromatic points^63^ among body regions (abdomen vs. dorsal region) and against the background (abdomen vs. background; dorsal region vs. background) using Avicol_v6^62^.

### Statistics

The R statistical package was used for analyses^68^. All data were checked for normal distribution using Shapiro-Wilk tests. Data that deviated from normal distribution were square-root transformed (chromatic contrast (abdomen vs. dorsal region) [egg-ripening cycle]; UV chroma [short-term response].

Using the “nlme” library, linear mixed effect models (“lme”) were conducted. In all performed models, the achieved chroma *r*_A_ and UV chroma as well as the calculated chromatic contrasts between abdomen, dorsal region and background served as dependent variable. “Individual” was included as random factor and never removed from the models to control for repeated measures. The different measuring points during the egg-ripening cycle (stripped, ripening, ripe) and for the short-term response the measurements before and after the animation (before, after) were used as explanatory variables. Furthermore, for the three measuring points during the egg-ripening cycle, post-hoc pairwise comparisons were conducted. Tests of significance were based on likelihood-ratio tests (LRT) that follow a χ^2^-distribution. P-values are two-tailed, and the α level set at 0.05.

## Results

### (A) Egg-ripening cycle

In the abdominal region, females differed significantly with respect to achieved chroma *r*_A_ (“lme”, N = 16; χ^2^ = 10.116, df = 2, p = 0.006) and UV chroma (“lme”, N = 16, χ^2^ = 41.675, df = 2, p < 0.001) over the complete course of the egg-ripening cycle (stripped, ripening, ripe). Pairwise comparisons between the measuring points “stripped” and “ripening” revealed a significantly enhanced UV chroma during the ripening process (Table 1). The achieved chroma *r*_A_ and the UV chroma were furthermore significantly higher in “ripe” females than in “stripped” females (Table 1). Comparing “ripening” and “ripe”, achieved chroma *r*_A_ and UV chroma were significantly higher in ripe females (Table 1).

**Figure 4:**
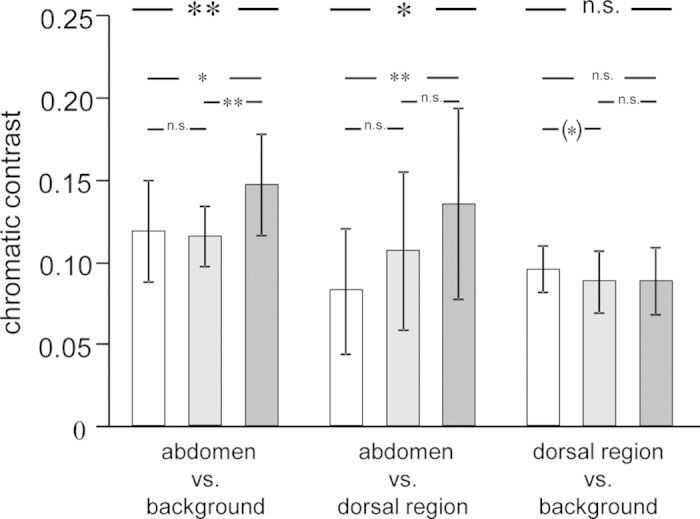
Chromatic contrasts between body regions and background during egg-ripening cycle. Shown are chromatic contrasts (abdomen vs. background, abdomen vs. dorsal region and dorsal region vs. background) of females during the egg-ripening cycle after being stripped (white bars), during the ripening process (light gray bars) and finally when ripe again (dark gray bars). Plotted are means and standard deviations. n.s. p > 0.10; (*): 0.05 < p < 0.10; *p < 0.05; **p < 0.01.

Chromatic contrast between abdomen and background (“lme”, N = 16; χ^2^ = 11.912, df = 2, p = 0.003; Fig. 4) and between abdomen and dorsal region (“lme”, N = 16; χ^2^ = 9.143, df = 2, p = 0.010; Fig. 4) differed significantly between the different stages of the egg-ripening cycle. The chromatic contrast between the dorsal region and the background did not differ significantly between stages (“lme”, N = 16; χ^2^ = 2.574, df = 2, p = 0.276; Fig. 4). Pairwise comparisons showed the chromatic contrast between abdomen and background was significantly higher in ripe females than in stripped or ripening females (Table 1; Fig. 4). Furthermore, chromatic contrast between abdomen and the dorsal region was significantly higher in ripe females than in stripped females (Table 1; Fig. 4).

### (B) Short-term response

The achieved chroma *r*_A_ as well as the UV chroma of females was significantly higher after being exposed to the standardized computer-animated reproductive male than before (Table 2; Fig. 2).

**Figure 5:**
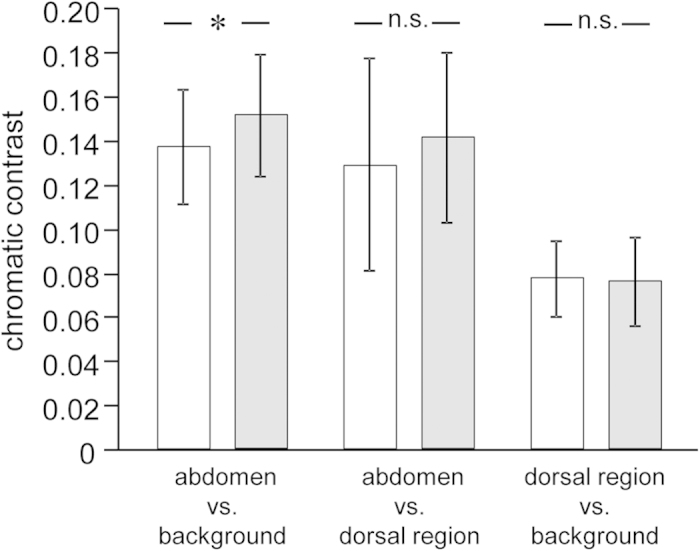
Chromatic contrasts between body regions and background as short-term response. Shown are chromatic contrasts (abdomen vs. background, abdomen vs. dorsal region and dorsal region vs. background) of females before (white bars) and after (light gray bars) presentation of a computer animation of a reproductively active male. Plotted are means and standard deviations. n.s. p > 0.10; *p < 0.05.

The chromatic contrast between the abdominal region and the experimental background was significantly enhanced after the animation (Table 2; Fig. 5), while the chromatic contrast between the abdomen and dorsal region as well as between the dorsal region and the background did not change significantly after the presentation of the male stimulus (Table 2; Fig. 5).

## Discussion

The present study shows that the intensity of UV reflections in the abdominal region of female sticklebacks varied considerably during the course of the egg-ripening cycle. The two measures used for color intensity in the UV spectral range, achieved chroma *r*_A_ and UV chroma, significantly increased throughout the ripening process and reached their maximum expression when females were ripe. A similar pattern was shown for the short-term response: achieved chroma *r*_A_ and UV chroma were significantly enhanced after the animation with a standardized reproductively-active courting male. UV coloration has previously been shown to be important in male mate-choice decisions in sticklebacks^36^. The varying intensities of abdominal UV reflections, peaking when females were ripe, hint at a role of UV in signaling female fecundity and readiness to spawn. There exist several possibilities of enhancing conspicuousness for conspecifics; either the UV reflection itself, contrast among ornaments or contrast against the background serves to enhance conspicuousness^69^. Studies on sticklebacks have shown that contrast among ornaments^70^ and against the background^24^ serve to increase conspicuousness in social contexts. In the present study, the results of the comparisons of chromatic contrast indicate that color contrast between the distended abdomen and the visual background is an important factor in female signaling. A higher contrast was detected for the abdomen against the experimental background (during the course of the egg-ripening cycle and as short-term response) and against the darker dorsal region (only over the course of the egg-ripening cycle) when females were ripe respectively had encountered a potential mating partner. The increase in color intensity in the UV spectral range respectively the contrast with the visual background most likely serves to attract males, thus underlining the importance of male mate-choice in this species^24^,^33^,^36^,^71^.

In the present study, we found evidence for seasonal as well as ephemeral color changes in the UV spectral range. Seasonal changes in UV reflections have, for example, been shown in Balkan moor frogs^48^. Furthermore, an influence of season has been reported in blue tits^47^; the authors found the blue tit UV/blue ornament to vary over the course of a year, also proposing a function in social signaling. Furthermore, Ornborg *et al.*^47^ found substantial variation in the structural color signal between populations. Population differences with regard to UV reflections of the female color signal in the abdominal region can be found in three spine sticklebacks as well, as reflectance measurements of German freshwater sticklebacks revealed a different spectral composition^36^. However, reflectance in the UV spectral range was generally found in all tested populations. The change in UV reflections after being confronted with the male animation was accomplished quite fast in the present study (less than 10 minutes). However, that lies well within the range of structural color changes that have been reported to take from one second only^13^ to several seconds or minutes^12^,^72^. Changes in structural coloration elicited by altered properties in iridophores have been shown frequently^11^,^12^,^14^,^15^,^16^. However, only very few studies have shown ephemeral changes with respect to UV coloration^12^,^13^.

In summary, UV reflections have been found to be highly dynamic in reproductively active female sticklebacks suggesting that changes in UV coloration seem to play an important role during courtship and in finding mating partners. We provide evidence that UV signals in females are dynamic throughout a reproductive cycle and as short-term response in an intersexual context.

## Additional Information

**How to cite this article**: Hiermes, M. *et al.* Context-dependent dynamic UV signaling in female three spine sticklebacks. *Sci. Rep.* **5**, 17474; doi: 10.1038/srep17474 (2015).

## Figures and Tables

**Table 1:** Results of the pairwise analysis of female color variables during the egg-ripening cycle.

Dependent variable	Explanatory variable	χ^2^	df	p	First point of time		Second point of time	
					*mean*	*SD*	*mean*	*SD*
achieved chroma *r*_A_ (abdomen)	stripped vs. ripening	0.585	1	0.444	0.124 ± 0.042		0.140 ± 0.074	
UV chroma (abdomen)		21.64	1	**<0.001**	0.220 ± 0.038		0.298 ± 0.043	
CC (abdomen vs. background)		0.137	1	0.711	0.119 ± 0.031		0.116 ± 0.019	
CC (abdomen vs. dorsal region)		2.576	1	0.109	0.083 ± 0.039		0.107 ± 0.048	
CC (dorsal region vs. background)		2.743	1	*0.098*	0.096 ± 0.014		0.089 ± 0.019	
achieved chroma *r*_A_ (abdomen)	stripped vs. ripe	4.459	1	**0.035**	0.124 ± 0.042		0.195 ± 0.072	
UV chroma (abdomen)		6.109	1	**0.014**	0.220 ± 0.038		0.339 ± 0.047	
CC (abdomen vs. background)		6.395	1	**0.011**	0.119 ± 0.031		0.148 ± 0.031	
CC (abdomen vs. dorsal region)		9.322	1	**0.002**	0.083 ± 0.039		0.136 ± 0.058	
CC (dorsal region vs. background)		1.394	1	0.238	0.096 ± 0.014		0.089 ± 0.021	
achieved chroma *r*_A_ (abdomen)	ripening vs. ripe	10.287	1	**0.001**	0.140 ± 0.074		0.195 ± 0.072	
UV chroma (abdomen)		35.659	1	**<0.001**	0.298 ± 0.043		0.339 ± 0.047	
CC (abdomen vs. background)		10.794	1	**0.001**	0.116 ± 0.019		0.148 ± 0.031	
CC (abdomen vs. dorsal region)		2.374	1	0.123	0.107 ± 0.048		0.136 ± 0.058	
CC (dorsal region vs. background)		0.007	1	0.935	0.089 ± 0.019		0.089 ± 0.021	

Given are achieved chroma *r*_*A*_, UV chroma and chromatic contrasts (CC) (abdomen vs. background; abdomen vs. dorsal region; dorsal region vs. background) calculated from reflectance spectra from females’ abdomen at three points of time during the female egg-ripening cycle: 1) female stripped of eggs (stripped), 2) throughout the ripening process (ripening) and 3) ripe females (ripe). Tests of significance were based on likelihood-ratio tests (LRT) that follow a χ^2^-distribution, hence, degrees of freedom always differed by one. Significant results (p < 0.05) are printed in bold, tendencies (0.05 < p < 0.10) are printed in italics. SD = standard deviation. N = 16.

**Table 2:** Results of the analysis of female color variables before and after the presentation of a computer animation.

dependent variable	explanatory variable	χ^2^	df	p	before animation		after animation	
					mean	SD	mean	SD
achieved chroma *r*_*A*_ (abdomen)	before vs. after	4.113	1	**0.043**	0.175 ± 0.061		0.204 ± 0.065	
UV chroma (abdomen)		7.308	1	**0.007**	0.330 ± 0.038		0.351 ± 0.042	
CC (abdomen vs. background)		6.406	1	**0.011**	0.138 ± 0.026		0.152 ± 0.028	
CC (abdomen vs. dorsal region)		1.328	1	0.250	0.130 ± 0.048		0.142 ± 0.039	
CC (dorsal region vs. background)		0.147	1	0.701	0.078 ± 0.017		0.077 ± 0.020	

Given are achieved chroma *r*_*A*_, UV chroma and chromatic contrasts (CC) (abdomen vs. background; abdomen vs. dorsal region; dorsal region vs. background) calculated from reflectance spectra taken at females’ abdomen before and after being animated by a computer animation of a reproductively active male. The models were compared with likelihood-ratio-tests (LRT) that follow a χ^2^-distribution. Significant results (p < 0.05) are printed in bold. SD = standard deviation. N = 23.

## References

[b1] Andersson M. Sexual selection. (Princeton University Press, 1994).

[b2] Bagnara J. T. & Hadley M. E. Chromatophores and color change: the comparative physiology of animal pigmentation. (Prentice-Hall, 1973).

[b3] Fujii R. The regulation of motile activity in fish chromatophores. Pigment Cell Research 13, 300–319 (2000). 11041206 10.1034/j.1600-0749.2000.130502.x

[b4] Bedizon Malek T. The genetic basis of male nuptial color in the threespine stickleback, Gasterosteus aculeatus. (ProQuest, 2008).

[b5] Stuart-Fox D. M. & Moussalli A. Camouflage, communication and thermoregulation: lessons from colour changing organisms. Philosophical Transactions of the Royal Society B 364, 463–470 (2009). 10.1098/rstb.2008.0254 PMC2674084 19000973

[b6] Kodric-Brown A. Sexual dichromatism and temporary color changes in the reproduction of fishes. American Zoologist 38, 70–81 (1998).

[b7] Sugimoto M. Morphological color changes in fish: regulation of pigment cell density and morphology. Microscopy Research and Technique 58, 496–503 (2002). 12242707 10.1002/jemt.10168

[b8] Sköld H. N. *et al.* Hormonal regulation of female nuptial coloration in a fish. Hormones and Behavior 54, 549–556 (2008). 18586039 10.1016/j.yhbeh.2008.05.018

[b9] Svensson P. A. *et al.* Temporal variability in a multicomponent trait: nuptial coloration of female two-spotted gobies. Behavioral Ecology 20, 346–353 (2009).

[b10] Ward J. L. & McLennan D. A. The relative influences of sexual and natural selection upon the evolution of male nuptial colouration in the brook stickleback, Culaea inconstans. Behaviour 143, 483–510 (2006).

[b11] Goda M. & Fujii R. The blue coloration of the common surgeonfish, *Paracanthurus hepatus* - II. Color revelation and color changes. Zoological Science 15, 323–333 (1998). 18465994 10.2108/zsj.15.323

[b12] Kasukawa H., Oshima N. & Fujii R. Mechanism of light reflection in blue damselfish motile iridophore. Zoological Science 4, 243–257 (1987).

[b13] Mäthger L. M., Land M. F., Siebeck U. E. & Marshall N. J. Rapid colour changes in multilayer reflecting stripes in the paradise whiptail, *Pentapodus paradiseus*. Journal of Experimental Biology 206, 3607–3613 (2003). 12966052 10.1242/jeb.00599

[b14] Oshima N., Kasukawa H. & Fujii R. Control of chromatophore movements in the blue-green damselfish, Chromis viridis. Comparative Biochemistry and Physiology C 93, 239–245 (1989).

[b15] Rohrlich S. T. Fine structural demonstration of ordered arrays of cytoplasmic filaments in vertebrate iridophores: a comparative survey. Journal of Cell Biology 62, 295–304 (1974). 4139162 10.1083/jcb.62.2.295 PMC2109405

[b16] Yoshioka S. *et al.* Mechanism of variable structural colour in the neon tetra: quantitative evaluation of the Venetian blind model. Journal of the Royal Society Interface 8, 56–66 (2010). 10.1098/rsif.2010.0253 PMC3024824 20554565

[b17] Chan R., Stuart-Fox D. & Jessop T. S. Why are females ornamented? A test of the courtship stimulation and courtship rejection hypotheses. Behavioral Ecology 20, 1334–1342 (2009).

[b18] Cuadrado M. Body colors indicate the reproductive status of female common chameleons: experimental evidence for the intersex communication function. Ethology 106, 79–91 (2000).

[b19] Heinsohn R., Legge S. & Endler J. A. Extreme reversed sexual dichromatism in a bird without sex role reversal. Science 309, 617–619 (2005). 16040708 10.1126/science.1112774

[b20] Stuart-Fox D. & Goode J. L. Female ornamentation influences male courtship investment in a lizard. Frontiers in Ecology and Evolution 2, 1–9 (2014).

[b21] Kekälainen J., Huuskonen H., Tuomaala M. & Kortet R. Both male and female sexual ornaments reflect offspring performance in a fish. Evolution 64, 3149–3157 (2010). 20629728 10.1111/j.1558-5646.2010.01084.x

[b22] Baldauf S. A., Bakker T. C. M., Kullmann H. & Thünken T. Female nuptial coloration and its adaptive significance in a mutual mate choice system. Behavioral Ecology 22, 478–485 (2011).

[b23] Berglund A. & Rosenqvist G. Male pipefish prefer ornamented females. Animal Behaviour 61, 345–350 (2001).

[b24] Rowland W. J., Baube C. L. & Horan T. T. Signaling of sexual receptivity by pigmentation pattern in female sticklebacks. Animal Behaviour 42, 243–249 (1991).

[b25] Svensson P. A., Forsgren E., Amundsen T. & Sköld H. N. Chromatic interaction between egg pigmentation and skin chromatophores in the nuptial coloration of female two-spotted gobies. Journal of Experimental Biology 208, 4391–4397 (2005). 16339859 10.1242/jeb.01925

[b26] Amundsen T. & Forsgren E. Male mate choice selects for female coloration in a fish. Proceedings of the National Academy of Sciences of the United States of America 98, 13155–13160 (2001). 11606720 10.1073/pnas.211439298 PMC60840

[b27] Greenwood A. K. *et al.* The genetic basis of divergent pigment patterns in juvenile threespine sticklebacks. Heredity 107, 155–166 (2011). 21304547 10.1038/hdy.2011.1 PMC3136628

[b28] Bakker T. C. M. & Mundwiler B. Female mate choice and male red coloration in a natural three-spined stickleback (*Gasterosteus aculeatus*) population. Behavioral Ecology 5, 74–80 (1994).

[b29] Wedekind C., Meyer P., Frischknecht M., Niggli U. A. & Pfander H. Different carotenoids and potential information content of red coloration of male three-spined stickleback. Journal of Chemical Ecology 24, 787–801 (1998).

[b30] Rowland W. J. Proximate determinants of stickleback behaviour: an evolutionary perspective. In The evolutionary biology of the threespine stickleback (eds. Bell A. M. & Foster S. A. ). 297–344 (Oxford University Press, 1994).

[b31] Rowland W. J. Mate choice by male sticklebacks, Gasterosteus aculeatus. Animal Behaviour 30, 1093–1098 (1982).

[b32] ter Pelkwijk J. J. & Tinbergen N. Eine reizbiologische Analyse einiger Verhaltensweisen von *Gasterosteus aculeatus* L. Zeitschrift für Tierpsychologie 1, 103–200 (1937).

[b33] Bakker T. C. M. & Rowland W. J. Male mating preference in sticklebacks: effects of repeated testing and own attractiveness. Behaviour 132, 935–949 (1995).

[b34] Rowland W. J. The ethological basis of mate choice in male threespine sticklebacks, Gasterosteus aculeatus. Animal Behaviour 38, 112–120 (1989).

[b35] Rowland W. J. & Sevenster P. Sign stimuli in the threespine stickleback (*Gasterosteus aculeatus*): a re-examination and extension of some classic experiments. Behaviour 93, 241–257 (1985).

[b36] Rick I. P. & Bakker T. C. M. UV wavelengths make female three-spined sticklebacks (*Gasterosteus aculeatus*) more attractive for males. Behavioral Ecology and Sociobiology 62, 439–445 (2008).

[b37] Rowe M. P., Baube C. L., Loew E. R. & Phillips J. B. Optimal mechanisms for finding and selecting mates: how threespine stickleback (*Gasterosteus aculeatus*) should encode male throat colors. Journal of Comparative Physiology A 190, 241–256 (2004). 10.1007/s00359-004-0493-8 14752565

[b38] Rick I. P., Modarressie R. & Bakker T. C. M. Male three-spined sticklebacks reflect in ultraviolet light. Behaviour 141, 1531–1541 (2004).

[b39] Rick I. P. & Bakker T. C. M. Color signaling in conspicuous red sticklebacks: do ultraviolet signals surpass others? BMC Evolutionary Biology 8, 189 (2008). 18593461 10.1186/1471-2148-8-189 PMC2453139

[b40] Rick I. P., Modarressie R. & Bakker T. C. M. UV wavelengths affect female mate choice in three-spined sticklebacks. Animal Behaviour 71, 307–313 (2006).

[b41] McLennan D. A. & McPhail J. D. Experimental investigations of the evolutionary significance of sexually dimorphic nuptial coloration in *Gasterosteus aculeatus* L.: temporal changes in the structure of the male mosaic signal. Canadian Journal of Zoology 67, 1767–1777 (1989).

[b42] Sevenster P. A causal study of a displacement activity (fanning in *Gasterosteus aculeatus* L.). Behaviour Supplement 9, 1–170 (1961).

[b43] Candolin U. Changes in expression and honesty of sexual signalling over the reproductive lifetime of sticklebacks. Proceedings of the Royal Society B 267, 2425–2430 (2000). 11133033 10.1098/rspb.2000.1301 PMC1690825

[b44] Cronly-Dillon J. & Sharma S. C. Effect of season and sex on the photopic spectral sensitivity of the three-spined stickleback. Journal of Experimental Biology 49, 679–687 (1968). 5702059 10.1242/jeb.49.3.679

[b45] Boulcott P. & Braithwaite V. A. Colour perception in three-spined sticklebacks: sexes are not so different after all. Evolutionary Ecology 21, 601–611 (2007).

[b46] Shao Y. T. *et al.* Androgens increase Iws opsin expression and red sensitivity in male three-spined sticklebacks. PLoS ONE 9, e100330 (2014). 24963891 10.1371/journal.pone.0100330 PMC4070989

[b47] Ornborg J., Andersson S., Griffith S. C. & Sheldon B. C. Seasonal changes in a ultraviolet structural colour signal in blue tits, Parus caeruleus. Biological Journal of the Linnean Society 76, 237–245 (2002).

[b48] Ries C., Spaethe J., Sztatecsny M., Strondl C. & Hoedl W. Turning blue and ultraviolet: sex-specific colour change during the mating season in the Balkan moor frog. Journal of Zoology 276, 229–236 (2008).

[b49] Stuart-Fox D. & Moussalli A. Selection for social signalling drives the evolution of chameleon colour change. PLoS Biology 6, e25 (2008). 18232740 10.1371/journal.pbio.0060025 PMC2214820

[b50] Delhey K., Burger C., Fiedler W. & Peters A. Seasonal changes in colour: a comparison of structural, melanin- and carotenoid-based plumage colours. PLoS ONE 5, e11582 (2010). 20644723 10.1371/journal.pone.0011582 PMC2904367

[b51] White T. E., Zeil J. & Kemp D. J. Signal design and courtship presentation coincide for highly biased delivery of an iridescent butterfly mating signal. Evolution 69, 14–25 (2014). 25328995 10.1111/evo.12551 PMC4312914

[b52] Rick I. P. & Bakker T. C. M. Ultraviolet light influences habitat preferences in a fish under predation risk. Evolutionary Ecology 24, 25–37 (2010).

[b53] Künzler R. & Bakker T. C. M. Female preferences for single and combined traits in computer animated stickleback males. Behavioral Ecology 12, 681–685 (2001).

[b54] Mazzi D., Künzler R. & Bakker T. C. M. Female preference for symmetry in computer-animated three-spined sticklebacks, Gasterosteus aculeatus. Behavioral Ecology and Sociobiology 54, 156–161 (2003).

[b55] Milinski M. & Bakker T. C. M. Female sticklebacks use male coloration in mate choice and hence avoid parasitized males. Nature 344, 330–333 (1990).

[b56] Künzler R. & Bakker T. C. M. Computer animations as a tool in the study of mating preferences. Behaviour 135, 1137–1159 (1998).

[b57] Mehlis M., Bakker T. C. M. & Frommen J. G. Smells like sib spirit: kin recognition in three-spined sticklebacks (*Gasterosteus aculeatus*) is mediated by olfactory cues. Animal Cognition 11, 643–650 (2008). 18465154 10.1007/s10071-008-0154-3

[b58] Richter L. Rotwahrnehmung und Partnerwahl beim Dreistachligen Stichling (*Gasterosteus aculeatus*): Einleitung, Material und Methoden. Project report. Bonn, University of Bonn. (2012).

[b59] Fleishman L. J., McClintock W. J., D’Eath R. B., Brainard D. H. & Endler J. A. Colour perception and the use of video playback experiments in animal behaviour. Animal Behaviour 56, 1035–1040 (1998). 9790716 10.1006/anbe.1998.0894

[b60] Gomez D. *et al.* The role of nocturnal vision in mate choice: females prefer conspicuous male in the European tree frog (*Hyla arborea*). Proceedings of the Royal Society B 276, 2351–2358 (2009). 19324736 10.1098/rspb.2009.0168 PMC2690462

[b61] Govardovskii V. I., Fyhrquist N., Reuter T., Kuzmin D. G. & Donner K. In search of the visual pigment template. Visual Neuroscience 17, 509–528 (2000). 11016572 10.1017/s0952523800174036

[b62] Gomez, D. *AVICOL, a program to analyse spectrometric data*. (2006) Available at: http://sites.google.com/site/avicolprogram/ or from the author at dodogomez@yahoo.fr. (Accessed: 8th July 2015).

[b63] Endler J. A. & Mielke P. W. Comparing entire colour patterns as birds see them. Biological Journal of the Linnean Society 86, 405–431 (2005).

[b64] Rick I. P., Mehlis M. & Bakker T. C. M. Male red ornamentation is associated with female red sensitivity in sticklebacks. PLoS ONE 6, e25554 (2011). 21984930 10.1371/journal.pone.0025554 PMC3184158

[b65] Stoddard M. C. & Prum R. O. Evolution of avian plumage color in a tetrahedral color space: a phylogenetic analysis of New World buntings. The American Naturalist 171, 755–776 (2008). 10.1086/587526 18419340

[b66] Drobniak S. M., Dyrcz A., Sudyka J. & Cichon M. Continuous variation rather than specialization in the egg phenotypes of cuckoos (*Cuculus canorus*) parasitizing two sympatric reed warbler species. PLoS ONE 9, e106650 (2014). 25180796 10.1371/journal.pone.0106650 PMC4152305

[b67] Shawkey M. D., Hill G. E., McGraw K. J., Hood W. R. & Huggins K. An experimental test of the contributions and condition dependence of microstructure and carotenoids in yellow plumage coloration. Proceedings of the Royal Society B 273, 2985–2991 (2006). 17015356 10.1098/rspb.2006.3675 PMC1639519

[b68] R-Development-Core-Team. *R: a language and environment for statistical computing*. (R foundation for statistical computing, 2009).

[b69] Endler J. A. On the measurement and classification of color in studies of animal color patterns. Biological Journal of the Linnean Society 41, 315–352 (1990).

[b70] Rush V. N., McKinnon J. S., Abney M. A. & Sargent R. C. Reflectance spectra from free-swimming sticklebacks (*Gasterosteus*): social context and eye-jaw contrast. Behaviour 140, 1003–1019 (2003).

[b71] Kraak S. B. M. & Bakker T. C. M. Mutual mate choice in sticklebacks: attractive males choose big females, which lay big eggs. Animal Behaviour 56, 859–866 (1998). 9790696 10.1006/anbe.1998.0822

[b72] Lythgoe J. N. & Shand J. Changes in spectral reflections from the iridophores of the neon tetra. Journal of Physiology 325, 23–34 (1982). 7108777 10.1113/jphysiol.1982.sp014132 PMC1251376

