# Erratum: Context-dependent dynamic UV signaling in female threespine sticklebacks

**DOI:** 10.1038/srep32903

**Published:** 2016-09-08

**Authors:** Meike Hiermes, Theo C. M. Bakker, Marion Mehlis, Ingolf P. Rick

Scientific Reports 5: Article number: 1747410.1038/srep17474; published online: 12 10 2015; updated: 09 08 2016

In the original version of this Article, “threespine stickleback” was incorrectly given as “three spine stickleback”.

This error has now been corrected in the PDF and HTML versions of the Article.

